# Supplementary figures and images for: Intracellular neutralisation of rotavirus by VP6-specific IgG
Source: PLoS Pathog. 2020 Aug 4;16(8):e1008732. doi: 10.1371/journal.ppat.1008732 (PMC7428215; doi:10.1371/journal.ppat.1008732)

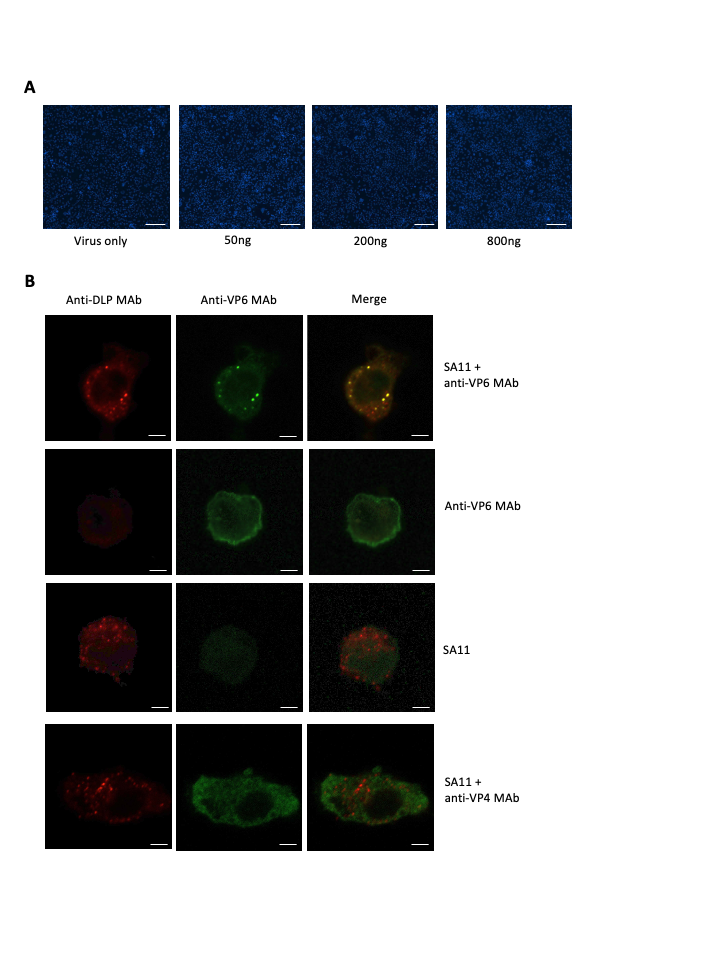

Supplement: S1 Fig — A) Nuclei stained with Hoechst 33342 from the 4 different wells presented in Fig 1A, scale bar 200μm. B) Extended Fig 1C showing additional controls used for confocal images; only in the presence of both SA11 rotavirus and anti-VP6 Mab is co-localization observed. Scale bar 10μm. (TIFF) [file ppat.1008732.s001.tiff]

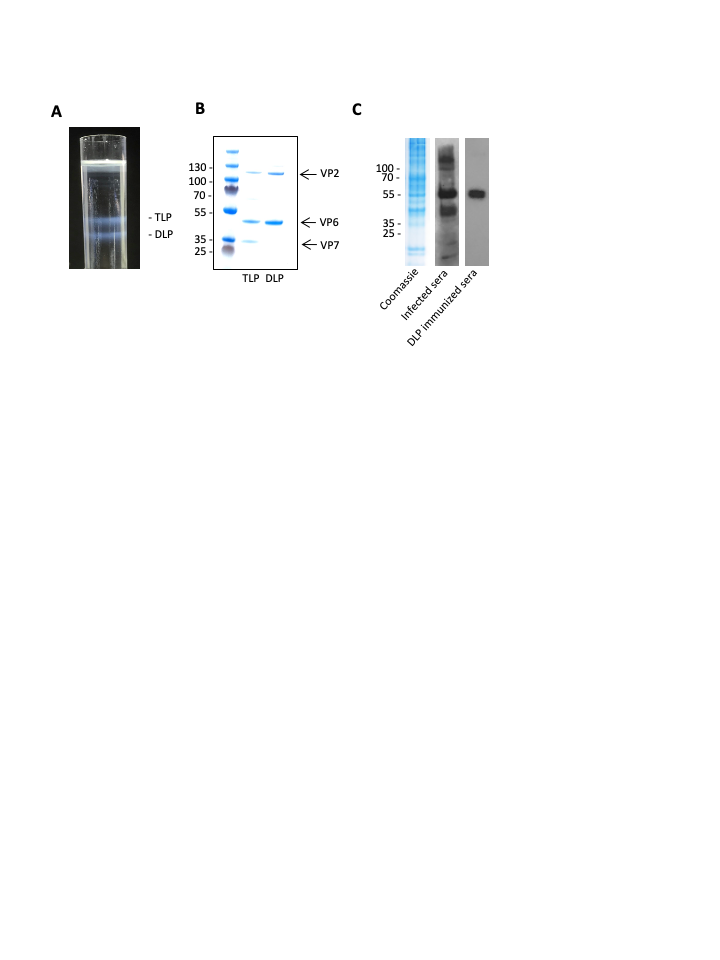

Supplement: S2 Fig — A) Image of viral bands after centrifugation at 110,000 x g for 18 hours on a CsCl gradient. B) Coomassie blue stained agarose gel of viral bands collected separately by needle puncture, demonstrating different protein composition of triple layered particles (TLP) and double layered particles (DLP). C) Western blot confirmation of the specificity of antibodies in sera from mice immunized with DLP in comparison with antibodies generated by mice infected with EDIM rotavirus; MA104 cells infected with rotavirus for 16 hours were lysed in SDS-PAGE loading buffer, the lysate separated by SDS-PAGE, then stained with Coomassie blue or western blotted with mouse sera. (TIFF) [file ppat.1008732.s002.tiff]

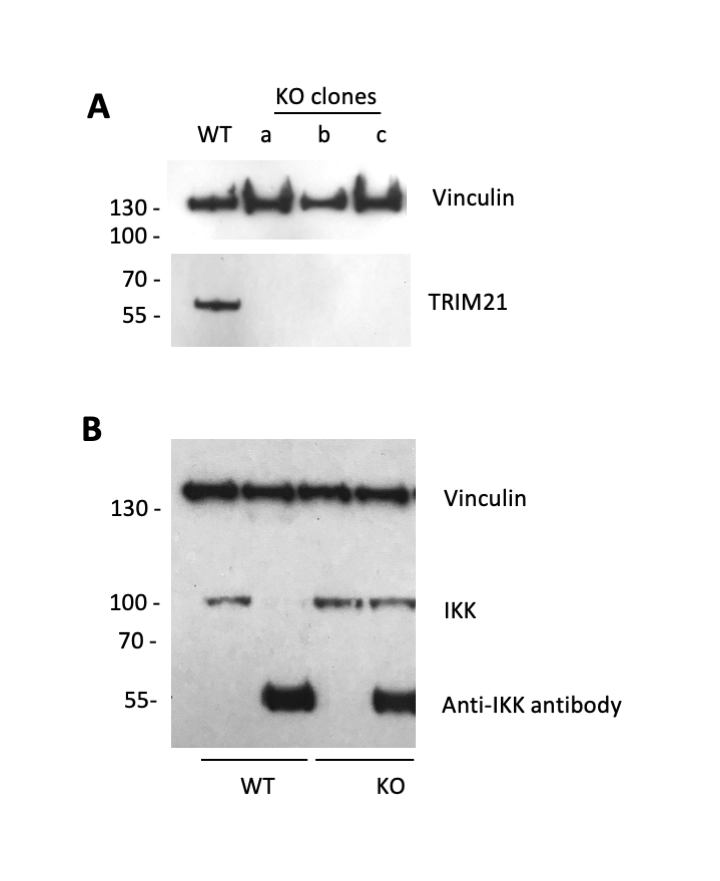

Supplement: S3 Fig — A) Western blot of whole cell lysate of one wild type (WT) MA104 clone and three TRIM21 knockout (KO) MA104 clones. B) Western blot showing ability of WT cells to degrade IKK when anti-IKK antibody is electroporated into cells (‘Trim-Away’), whereas no degradation of IKK is mediated by TRIM21 KO cells (clone b). (TIFF) [file ppat.1008732.s003.tiff]

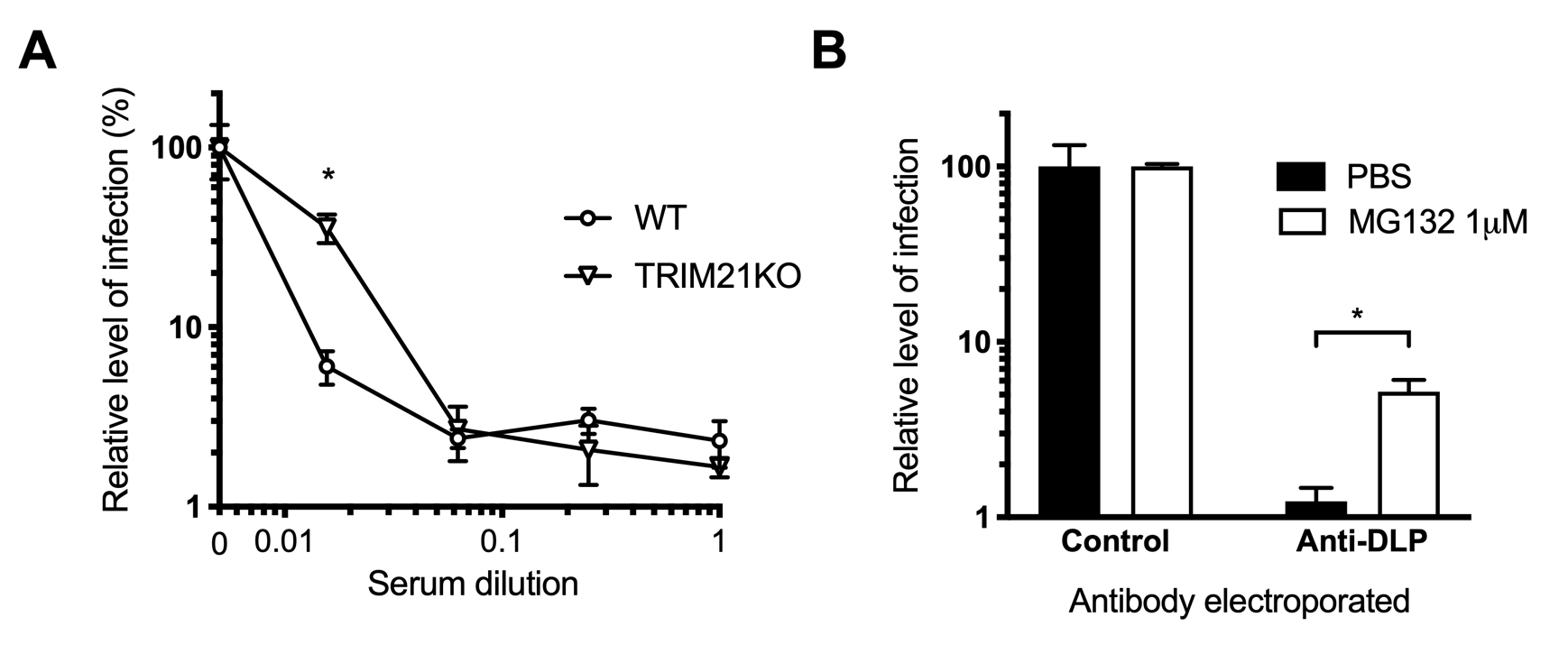

Supplement: S4 Fig — A) Intracellular neutralisation in wild type (WT) and TRIM21 knock out (TRIM21KO) cells by serially diluted anti-DLP polyclonal serum. B) Intracellular neutralisation of rotavirus by undiluted anti-DLP polyclonal serum and control serum in the presence of 1μM MG132. For both graphs, error bars represent standard error, * p = < 0.05. (TIFF) [file ppat.1008732.s004.tiff]
