# Supplementary material for: Intracellular neutralisation of rotavirus by VP6-specific IgG
Source: PLoS Pathog. 2020 Aug 4;16(8):e1008732. doi: 10.1371/journal.ppat.1008732 (PMC7428215; doi:10.1371/journal.ppat.1008732)
Supplement: Attachment — Submitted filename: Caddy et al, reply rev 7-4-20.pdf [file ppat.1008732.s005.pdf]

Response to reviewers' comments

Reviewer #1: *Major points:*
1) In Fig. 1A, is there a reason why "fluorescent focus forming reduction assay" (Results, Line 350) was used as opposed to traditional plaque forming unit assays?

We chose to use the fluorescent focus forming reduction assay to study viral titres as this approach has been shown to be comparable to results provided by traditional plaque assay (Yang et al, Clin Diagn Lab Immunol. **1998** Nov;5(6):780-3), yet provides more rapid results (16 hours v 3-4 days). Furthermore, this type of quantification allowed us to develop a high-throughput method using the high content Nikon Eclipse Ti microscope.

One potential caveat of this imaging-based assay is that the anti-VP6 antibodies may compete with the detection antibody, giving rise to false positive results.

We used a sheep polyclonal anti-rotavirus antibody that targets all viral proteins as our detection reagent. Even if intracellular anti-VP6 antibodies reduce polyclonal antibody binding to VP6, antibodies specific for VP2, VP4 and VP7 will still identify infected cells.

Also, viral antigens were stained at 16 hours post infection (Materials and Methods, Line 155). The authors are encouraged to perform plaque assays within a single replication cycle (8 hours) to determine the neutralization capacity of these anti-VP6 IgGs.

As plaque formation usually requires more than one round of replication, a plaque assay at 8 hours is unlikely to give a strong signal. However, we are grateful for the suggestion to look at neutralization kinetics and plan in future work to use recently generated reporter-tagged rotavirus (Kanai Y et al, *Proc Natl Acad Sci USA* **2017** Feb 28;114(9):2349-2354; Philip AA, et al, *Microbiol Resour Announc.* 2019 Jul 3;**8**(27). pii: e00523-19) and follow viral replication in live cells.

2) It is stated in Materials and Methods, Line 210, that CsCl gradient ultracentrifugation was used to purify double-layered particles (DLPs), which were later used for immunization studies (Fig. 2). Did the authors test infectivity of their DLP preparation? It is possible that a trace amount of triple-layered particle contaminants induced anti-VP4 and VP7 antibodies. Have the authors tested their anti-DLP sera by western blot or ELISA to rule out immunity to either VP4 or VP7?

We agree that low levels of triple-layered particle contaminants are possible when DLPs are purified on CsCl gradients, hence we were very careful to confirm the purify of the preparations we generated. We have now included this data as supplementary figure 2, which demonstrates the high level of purity in our DLP preparation by Coomassie blue staining of DLP and TLP bands separated by SDS-PAGE.

We followed the suggestion to directly test the antibody response in our anti-DLP serum by western blot. We have now performed this experiment and include the data in

supplementary figure 2c. When sera from mice challenged with EDIM rotavirus were compared with sera from mice immunized with DLPs, it is clear to see that DLP immunization only resulted in detectable production of anti-VP6 antibodies.

3) Data in Fig. 3 is not compelling and can be contributed by antibody binding affinities. For instance, at 103 ng/ml, 7D9 hIgG reached an OD of 3.0 (Fig. 4A) whereas 7D9 hIgA was only at 1.2 (Fig. 4B). In theory, the reduced blocked of infectivity by hIgA could be due to a weaker binding rather than the Fc region (Fig. 4C). To definitely show that the Fc of IgG is required, the authors should generate a Fab fragment and demonstrate the loss of activity.

Detection of IgG and IgA by ELISA requires the use of different secondary antibodies, which means it is not possible to directly compare the OD450 values between graphs. Also, as these antibodies were produced by recombinant antibody technology, we are confident that isotype switching has not been accompanied by any changes in Fab sequence and therefore antigen binding affinity. While generating Fab fragments does remove the contribution of the Fc it also changes antigen binding from bivalent to monovalent, meaning that loss of avidity will also contribute to any reduction in neutralization. The data in Figure 3C distinguishes the contribution of an IgG Fc vs IgA Fc as the antigen binding component of both antibodies is the same. This result is also consistent with the published work on TRIM21 demonstrating that binding takes place solely to the Fc e.g. James et al 2007 [35], Mallery et al 2010 [19] and Bottermann et al [*Cell Host Microbe.* 2019 Apr 10;**25**(4):617-629.e7].

4) The authors should elaborate or at least speculate on the mechanism of intracellular IgG-mediated neutralization. Is it transcriptional inhibition by blocking the mRNA portals on DLPs or is it DLP degradation?

We appreciate the suggestion to discuss possible mechanisms of intracellular IgG neutralisation, and have amended our manuscript accordingly (lines 541-544). We discuss two potential mechanisms of neutralisation: inhibition of mRNA production by DLPs (as shown by Aiyegbo et al, 2013 [18], Thouvenin et al, 2001 [16], Kohli et al, 1994 [32]) and DLP degradation via VP6-specific ab-TRIM21 complex degradation (this manuscript). We have also included an additional supplementary figure (S4) that shows intracellular neutralisation is reduced in the presence of the proteasome inhibitor MG132, which supports the conclusion that TRIM21-mediated DLP degradation occurs via the proteasome.

5) It is hard to imagine that if anti-VP6 IgG is important for protection in humans, there will be no epidemiological data to support that. In the vaccinated children cohort, do anti-VP6 IgG levels correlate with protection?

There is indeed epidemiological data showing cross-protection, e.g. after immunization with Rotarix (monotypic G1P[8]) against G2P[4] wildtype rotaviruses (e.g. Ruiz-Palacios et al, *N Engl J Med* 2006; **354**: 11-22; Vesikari et al, *Lancet* 2007; **370:** 1757-1763), which is potentially mediated by VP6-specific antibody. Direct experimental evidence of the protective efficacy of anti-VP6 IgA has also been published by Burns et al, 1996 [8]. Thank you for the suggestion of studying anti-VP6 IgG levels in vaccinated children; this is planned for future work and we are awaiting the serum samples from a large clinical trial.

Reviewer #2: *1. The authors have presented data on effects of Ig on RV throughout the paper as a percentage effect (relative level of infection) on a 10-log scale. This is very confusing and hampers evaluation of claims on how potent the neutralization effects are likely to be. This reviewer suggests presenting the viral titers on a 10-log scale – or as a less clear alternative to show the relative percentages with errors on a linear scale.*

We have changed the way *in vitro* data are presented by showing relative percentages with errors on a linear scale. We agree this is a clearer way to show the significant differences between the different neutralisation conditions studied. It has been necessary to keep showing relative percentages instead of viral titre, as certain graphs present a combination of different assays (e.g. figure 1d - extracellular and intracellular neutralisation), or treatment affects viral titre (e.g. figure S4b – MG132 decreases viral replication). The only exception to presentation of data on linear scales is now is figure S4b, where the changes between neutralisation conditions are unclear on a 0-100% scale – we have emphasised in the associated text that the changes observed were small.

2. The statistical significance should be marked for every data panel – including in cases where there is no significant difference. The results and discussion should be in accordance with the statistical interpretation.

Thank you for this recommendation, all figures have now been updated accordingly. Associated comments have also been included in the results and discussion.

3. Figure legends are missing details to allow the reader to understand what was done in each experiment. This should be included.

Figure legends have now been extended, and we hope they are now fully understandable to any reader.

Reviewer #3: *Figure 1A. Electroporation is known to be toxic to cells. Was LDH or another method used to measure cell viability? Nuclei should be stained to show that the cell density is similar in each of the wells. What is the titer of virus at 100% infection? It is critical to determine the cytotoxicity of electroporation in all the electroporation experiments.*

In our study we utilised the Neon^{TM} electroporation system, which does not suffer the drawbacks of high current and concomitant cell death as 1st generation Amaxa devices. We employed the antibody delivery protocol established in Clift et al, Cell, 2017 [19]. In figure 5 of this study, we demonstrated that the antibody electroporation results in highly efficient delivery of antibody into the cytoplasm without cell death. Electroporated cells showed no evidence of stress or damage and rapidly re-entered the cell cycle with similar long-term division rates as non-electroporated cells.

To demonstrate that cell survival or cell density was not compromised following electroporation in the intracellular neutralisation assay for rotavirus, in the new supplementary figure S1A we have included images of cell nuclei stained with Hoechst 33342 as requested for each of the wells presented in main figure 1A.

The titre of the virus stocks used was 1 x 10^7 FFU/ml, and 1000FFU were added to each well of the 96-well plate as described in the materials and methods. This quantity of virus was carefully chosen to enable reliable counting of foci by the NIS analysis software.

The text indicates that a non-specific control antibody was also electroporated in this experiment. Was the amount of virus quantified in the control antibody electroporated cells? I'm assuming this would be indicated in Figure 1B.

A total of 800ng non-specific antibody (anti-adenovirus hexon 9C12) was electroporated into cells as a control for each assay. This quantity matched the highest amount of rotavirus-specific antibody electroporated. The number of foci in wells electroporated with VP6-specific antibody were then calculated relative to the number of foci in wells electroporated with non-specific control antibody. This has now been clarified in the text, lines 355 and 361-362.

Figure 1C. There can be a slow exchange of secondary antibody in these types of experiments since 2 different mouse antibodies were used for this staining. Were proper controls performed to show that the detection is specific?

Thank you for highlighting this potential issue. We have included an additional panel in supplementary figure 1 in order to show the extended range of controls conducted for this experiment. These include cells electroporated with VP6-specific antibody with no infection, SA11 infection only, and SA11 infection in the presence of VP4-specific antibody. No apparent colocalization between DLPs and antibody was observed for any of these control conditions.

In many of the figures error bars are not indicated. How many times were experiments repeated and how many technical replicates were performed within each experiment?

We apologise that this was not clear in the original figures; in some of the figures the error bars were too small to distinguish. To amend this we have made the size of each symbol smaller to allow the error bars to be seen more clearly. The only figure not to include error bars was figure 3C, which we have now corrected and we apologise for this oversight. Three technical replicates were performed for every experiment, and every experiment was performed at least twice.

Figure 2. It is very difficult to purify DLP that do not generate antibodies against the outer capsid proteins. How pure were the immunizing DLPs? Did the mice generate antibodies against VP4 or VP7? Was this tested by western blot or another method to determine whether the mice generated antibodies against VP4 or VP7?

We agree that this is an important consideration. We have performed the suggested western blot data and added this data as supplementary figure S2. DLP-immunization did not induce detectable VP4- or VP7-specific antibodies. [See reply to comment 2 of reviewer 1.]

Figure 2B. It has been shown that mice immunized with non-replicating virus-like particles composed of VP2 and VP6 are protected from challenge. The author's result with DLPs is expected. However, other animal models such as rabbits, pigs or cows are not protected from

disease or shedding following immunization with VP2/VP6 VLPs. It is only in mice that DLPs or VP2/VP6 VLPs is protective and this should be discussed.

While not all animal models will show protection after immunization with VP2/VP6 virus-like particles (VLPs), it is clear from human universal rotavirus vaccination data that neutralizing (NT) antibodies (directed against VP7 and VP4) provide only a partial correlate of protection. Since VP6-specific antibodies are the most abundant after both natural infection and vaccination (Svensson et al, 1987a, b [4, 5]), they are good candidates as a missing correlate of protection. It is precisely this gap in knowledge which prompted our investigation. We have expanded on this point as suggested and addressed the question of a failure in anti-DLP antibodies to provide protection in some animals in the discussion section in lines 548-552. [See also reply to comment 5 of reviewer 1].

Figure 2C. The amount of IgG vs IgA cannot be accurately determined by this ELISA. The amount of each antibody type has to be determined based on a standard curve.

Thank you for identifying this issue. We agree that the ELISA used does not enable the amount of each antibody isotype to be calculated. Our description of this data was inaccurate; it was intended to convey not the absolute quantity of each protein but whether there were anti-DLP antibodies of each isotype induced in the immunised animals. We have amended the text (Lines 390-391) to state that: "the mice immunized with DLPs and protected from EDIM produced a significant IgG anti-VP6 response (figure 2C). Conversely, no significant difference was observed between IgA titres in naïve and immunized mice.".

Figure 2D. What virus and cells were used for this experiment? This was not described in the figure legend or the text. Was a western blot used to verify that antibody against VP6 was the only antibody isolated with the DLPs conjugated to agarose beads?

Thank you for bringing this omission to our attention. As with all other *in vitro* experiments in our study, we infected MA104 cells with SA11 rotavirus. This has been clarified in the text. We did not test the VP6 antibody by western blot to show that it recognised VP6, as this was confirmed via intracellular neutralisation assay. As presented in figure 1E, if we used purified antibody specific for the outer capsid proteins (or any other non-rotavirus protein), no intracellular neutralisation occurred.

How was the TRIM21 knockout confirmed in the MA104 cells?

We used two methods to show that TRIM21 was knocked out in the MA104 cells lines. Firstly, the absence of TRIM21 expression was confirmed in three knockout clones relative to wild type using western blotting for TRIM21 protein. Secondly, to test the functional activity of the knockout cells we then performed a 'Trim-Away' experiment (first described by Clift et al, Cell, 2017, ref 29). We have now added supplementary figure S3 presenting these results in the manuscript.

Although significance is stated in the text for Figs. 4B and C, statistics are not shown on the figure.

We are unsure how to address this comment as significance is not stated in the text for figures 4B and C. If instead the reviewer is referring to figures 4D and E, then the levels of significance were already presented in figure 4D as *, with details in the legend. We have now added * to figure 4E as well.

If TRIM21 is the mechanism by which 7D9 IgG is mediating the neutralization, why are higher concentrations of the antibody able to neutralize either in the absence of TRIM21 or when the interacting amino acid is mutated?

It has previously been shown that 7D9 IgA can block mRNA egress through DLP pores (Feng et al 2002 [7]). As the binding affinity of 7D9 IgG is identical to IgA, we expect that the IgG isotype will also mediate this mechanism of neutralisation. However, the IgG Fc region enables additional activity of TRIM21, providing two distinct mechanisms of neutralisation. TRIM21 has shown to be effective when <2 antibodies bind to the surface of adenovirus (McEwan et al 2012, [20]), so we predict that TRIM21 activity is most apparent at low antibody concentration. This is supported by a new supplementary figure (S4A) that presents intracellular neutralisation mediated by DLP-specific serum. At higher levels of antibody, pore blockade becomes the dominant neutralisation method. An additional sentence has been added to the discussion to explain this reasoning.

Was a confirmatory experiment performed to show that introduction of the H433A mutation in 7D9 IgG abrogated the antibody interaction with TRIM21?

We agree that it is important to know that this mutation abrogates TRIM21 binding and we have included an additional sentence in the results (Line 441). This is in fact a well-characterised mutant and while we did not repeat the experiments here, we have previously shown that H433A abrogates IgG binding to TRIM21 (Foss et al. 2016 [31]). We have also shown that this loss of binding results in loss of TRIM21 interaction and activity in cells (McEwan et al 2012 [20] and Bottermann et al, *Cell Host Microbe* 2019, cited above).

For the experiments shown in Figs. 4D and E, were RV-specific antibody titers determined as well as the immunoglobulin type of the antibody?

RV-specific ELISAs for both IgA and IgG were performed for serum sample from mice immunized with DLPs as shown in figure 2C. Serum ELISAs were not performed for every WT and KO mice immunized with DLPs in figure 4, as both the dose and the batch of DLPs that were administered to the second group of mice were identical to that administered to the first group.

The figure legend for 4E is unclear. Does this represent EDIM shedding days 1-7 post infection? As it reads, the mice were immunized with DLPs on days 1-7 post infection. Are any of the time points significantly different?

Thank you for highlighting this issue with the legend. The mice were immunized with DLPs 28 days and 14 days prior to infection only. This has now been clarified in the materials and methods, in the results and in the legend itself.

In all the mouse experiments, how many mice were in each group? This should be stated in the figure legend although I'm assuming that the number of markers shown in the graph indicates a separate mouse. For figure 5A, it appears there are only 3 mice in the FcRn & DLP group.

For figures 2C, 4D and 5A/C, each point on the scatter plot represents one mouse. This is now clarified in each legend. As mentioned already in the text, in figure 5C there were 5 mice in the FcRn & DLP group, but only 3 mice had detectable levels of antibody. To make this clearer in the figure, all mice with OD450 < 0 (indicating blank wells had higher scores than antigen wells), are now presented as OD450 = 0.

In the discussion, the authors indicate that TRIM21 is activated when it binds the Fc portion of cytosolic antibodies bound to virus, catalysing auto-ubiquitination. This ubiquitination targets the virus-antibody complex to the proteasome for degradation. If this is the case, can the authors show that the electroporated VP6 mAb is degraded in MA104 cells and this is proteasome-dependent by the addition of MG132? In addition, the mAb should not be degraded in the TRIM21 KO MA104 cells. Is proteasomal degradation the fate of the DLPs in the electroporated cells? Is this the mechanism of "neutralization"?

Thank you for this suggestion, we agree that use of MG132 to inhibit the proteasome is an additional way to verify the role of TRIM21 in IgG-mediated intracellular neutralisation of rotavirus. The challenge we have in successfully conducting the experiment described is that in order to detect VP6 MAb by western blot we would need to electroporate much larger quantities of the MAb into cells. Unfortunately, the 7D9 IgG is a recombinant antibody and the current yields we obtain when producing it are too low.

Instead, we have performed an intracellular neutralisation experiment in the presence of MG132, and shown that neutralisation is impaired relative to treatment of cells with PBS. This is now presented in supplementary figure 4, supporting the known role of TRIM21 in recruiting the proteasome to drive virus particle degradation, as initially described by Mallery et al, 2010 [19]. We would have liked to extend this experiment with TRIM21KO cells and the panel of recombinant VP6-specific antibodies generated for this project, but because of lab closure due to the COVID-19 pandemic we have been unable to complete this plan. We do not feel, however, that this additional work would affect our conclusions, as we have been able to use multiple other approaches to show a role for TRIM21 in intracellular neutralisation of rotavirus.

Part III – Minor Issues: Editorial and Data Presentation Modifications

Reviewer #1: Minor points:
1) Abstract, Line 28, VP6 is the "middle" capsid protein, not the "inner" capsid protein.
This has been corrected.

2) Abstract, Line 35, there was no data to support that the neutralization is "in the cytoplasm" or endosome/membrane-associated.
It is known that TRIM21 is only present and active in the cytoplasm (Mallery et al, 2010 [19]), therefore as we have demonstrated a role for TRIM21-mediated virus neutralisation, we are confident this must be cytoplasmic. However we agree that we have not definitively shown this in this study, and have therefore amended line 35 in the abstract from 'in the cytoplasm' to 'inside cells'.

3) Introduction, Line 73, "Species A" should be "Group A".
We understand that species is the correct term, as used by Matthijnssens et al *Arch Virol.* 2012 Jun;**157**(6):1177-82.], although we agree that in the literature 'group' is often used; both terms describe the same parameter.

4) Materials and Methods, Lines 157 and 165, the subtitle should be in bold to be consistent with the other parts.
This has been changed.

5) Materials and Methods, Line 182, "electroporated into MEF cells". However, I cannot find the use of MEFs in the paper.
MEFs were mentioned in the Cells and Viruses section and used for confocal imaging as stated in the immunofluorescence section of Materials and Methods used.

6) Scale bars should be provided for Figs. 1A and 1C.
These have been added.

7) In Fig. 1D, the label for 7D9 extracellular should be circles instead of squares.
This has been changed.

8) The labels for Figs. 4A and B are very confusing. The x-axis should be made consistent with Ig classes: IgA for 4A and IgG for 4B.
Thank you for highlighting this, the x-axis labels have now been amended

9) In Fig. 4A, how were the TRIM21 KO MA104 cells validated? By western blot or Sanger sequencing? Please specify.
Validation was carried out by western blotting for protein expression. This is now clarified by the addition of supplementary figure S4 which confirms no TRIM21 protein is expressed in the KO cells compared to WT cells. We also demonstrated absence of any TRIM21 activity in these cells using a 'Trim-Away' experiment as explained in the revised manuscript.

10) Figs. 4D-E, do the authors have evidence that the effect of TRIM21 is T cell-independent?
We know that TRIM21 blocks virus independently of T cells as we have demonstrated that TRIM21-dependent neutralisation occurs *in vitro* in the absence of any T cells. However, we agree that we can't rule out the possibility that TRIM21 also has an additional function that promotes T cells *in vivo*. The only way to answer this question would be to repeat our *in vivo* experiments in mice depleted of T cells. However, a previous study of rotavirus infection in mice showed that T cells are not required for protection against re-infection with rotavirus (Franco MA, Greenberg HB. *J Virol.* 1995 Dec;**69**(12):7800-6). This suggests that in our DLP-immunization model TRIM21 is not acting via T-cells.

11) In Fig. 5A, what are the levels of DLP-specific IgG in the intestinal tissues?
Serum ELISAs were performed to study the levels of DLP-specific IgG. No intestinal tissue samples were examined.

12) There were no data to support that IgG actually get inside cells. To bridge the MA104 results with the FcRn KO mice data, can the authors generate an MA104-FcRn stable cell line, which would be more physiologically relevant than electroporation.
IgG antibodies continuously enter cells via micropinocytosis. It is the job of the neonatal receptor (FcRn) to recycle IgG-containing endosomes back to the plasma membrane or transcytose them across cells (Ghetie V, Ward ES, *Immunol Res.* 2002;**25**(2):97-113). The key question is therefore how antibodies get from endosomes into the cytosol. We hypothesise that it is the virus that carries the antibodies into the cytoplasm. It is widely accepted that VP6-specific IgAs encounter DLPs in endosomes, then IgA enters the cytoplasm bound to and transported by the DLPs as part of the normal viral life-cycle. We expect that IgGs are similarly transported from endosomes into the cytoplasm by DLPs. We have outlined this proposed mechanism in Lines 560-570. We are grateful for the suggestion to use a stable cell line that expresses FcRn. We now intend to compare transport of antibodies and viruses in transwell systems using tight monolayers of cells expressing either the polyIgR receptor or FcRn and compare IgA and IgG. However, we anticipate that this will be a significant study in its own right.

13) Figure legends, Lines 823 and 825, (B) and (C) were arranged in the wrong order.
The legend has now been corrected

14) Figure legends, Line 828, there was no panel (D) in the Fig. 5.
The legend has now been corrected

15) Statistics should be provided for all figure panels.
These have now been provided.
