# Supplementary material for: Intracellular neutralisation of rotavirus by VP6-specific IgG
Source: PLoS Pathog. 2020 Aug 4;16(8):e1008732. doi: 10.1371/journal.ppat.1008732 (PMC7428215; doi:10.1371/journal.ppat.1008732)
Supplement: Attachment — Submitted filename: Caddy et al letter to editor-2.pdf [file ppat.1008732.s006.pdf]

Leo James, Ph.D.

Division of Protein & Nucleic Acid Chemistry
MRC Laboratory of Molecular Biology
Francis Crick Avenue
Cambridge CB2 0QH

MRC | Laboratory of Molecular Biology

Dear Editors

Thank you for reviewers' comments on our revised manuscript entitled **'Intracellular neutralisation of rotavirus by VP6-specific IgG'** (PPathogens-2020-00106R1).

We are pleased that reviewer 1 was largely satisfied with our revision, and we have the following responses to their latest comments;

Reviewer #1: This is a revision of the original manuscript by Caddy SL et al. Specifically, the authors made extensive edits to the text and added new supplementary figures 1-4 to address the potential concerns. Most of the points raised were satisfactorily addressed. However, this reviewer finds the response to Question 1 insufficient. [In Fig. 1A, is there a reason why "fluorescent focus forming reduction assay" was used as opposed to traditional plaque forming unit assays?] Plaque assays unable to "provide more rapid results (16 hours v 3-4 days)" and "a highthroughput method using the high content Nikon Eclipse Ti microscope." seem irrelevant here.

Thank you for your comment, and apologies if we have misunderstood your original question regarding the use of plaque assays. We chose to use a focus forming assay (FFA)-based approach to study virus replication as the FFA is a standard test that is widely used in virology and shown to provide comparable results to plaque assays (Yang et al, Clin Diagn Lab Immunol. 1998 Nov;5(6):780-3). Our comment on the more rapid timescale provided by the FFA was because, all things being equal, a shorter timescale is a practical advantage.

Other alternative approaches such as focus forming unit assays and quantitative PCR are also readily available to confirm the authors' findings.

There may be some misunderstanding here but the assay we used **is** a focus forming unit assay, which is itself simply a variation of a plaque assay. Our assay uses immunostaining with fluorescently labelled antibodies specific for viral antigen instead of relying on cell lysis. We used the phrase 'reduction assay' to reflect the fact that we are using antibody-mediated neutralisation to reduce the number of foci forming.

We agree that qPCR is a readily available technique and a useful approach to probe viral biology. However, it measures RNA levels and not infectious viral progeny. The FFA we used is a more direct measure of virus replication.

The fact that "sheep polyclonal anti-rotavirus antibody that targets all viral proteins as our detection reagent" does not give support to the validity of the assay used in Fig. 1.

The read out for our intracellular neutralisation assay is simply infected cells, which is detected by the use of a secondary antibody that can detect a wide range of viral proteins. If electroporated antibodies were competing with the detection antibodies, then we would expect to see a comparable decrease in infection with both VP6 and VP4-specific antibodies, but we have demonstrated that only electroporation of VP6-specific antibodies results in a decrease in infected cells (Fig 1D).

Two minor points: 1) "dependant" should be "dependent" in marked document, lines 425 and 905;
This has been corrected as advised.

Tel: +44 (0)1223 267162 Email: lcj@mrc-lmb.cam.ac.uk Web: http://www2.mrc-lmb.cam.ac.uk/group-leaders/h-to-m/l-james

Statistics should be applied to Figs. 3A, 3C, 4A-C, and S4A-B.
These have been added to each figure, and/or discussed in the results section as advised.

Thank you for your consideration of this revision, and we hope that you will now find our revised manuscript suitable for publication.

Kind regards,

Sarah Caddy and Leo James
